# Exploring the Determinants of Antenatal Care Services Uptake: A Qualitative Study among Women in a Rural Community in Northern Ghana

**DOI:** 10.1155/2019/3532749

**Published:** 2019-12-18

**Authors:** Gilbert Ti-enkawol Nachinab, Charles Ampong Adjei, Florence Assibi Ziba, Richard Asamoah, Priscilla Adumoah Attafuah

**Affiliations:** ^1^Department of Midwifery, School of Allied Health Sciences, University for Development Studies, Tamale, Ghana; ^2^Department of Community Health Nursing, School of Nursing and Midwifery, University of Ghana, Legon, Ghana; ^3^Department of Nursing, School of Allied Health Sciences, University for Development Studies, Tamale, Ghana; ^4^University of Ghana Hospital, Legon, Ghana

## Abstract

**Background:**

Global evidence has shown significant contribution of Antenatal care (ANC) in the detection and treatment of pregnancy related complications. Over the years, many areas in Ghana have recorded high uptake of ANC. However, this is not the case for Binduri district in Northern Ghana where only 37.4% of pregnant women utilised the services of ANC during their period of pregnancy compared to a national figure of 87%. We therefore sought to explore the determinants of ANC uptake among women who failed to utilise ANC services during their period of pregnancy in Binduri District in Northern Ghana.

**Methodology:**

The study was an exploratory descriptive study using purposive sampling technique. A total of 15 women who met the inclusion criteria for the study were recruited for a face-to-face interview. The data were analysed using the procedure of inductive thematic analysis.

**Results:**

The study findings showed that several factors hindered the use of ANC among our participants. The individual factors that were responsible for nonutilisation of ANC included financial constraints hindering registration with the national health insurance scheme, excuses of being busy, perception that pregnancy was not sickness and concentration on work. Perceived poor attitude of nurses was the only health system factor that contributed to non utilisation of ANC services.

**Conclusion:**

There is the need for establishment of registration centres of the national health insurance in all communities to make the scheme more accessible. There should also be intensive public education on importance of attending ANC.

## 1. Introduction

Antenatal care (ANC) is the care given to women during pregnancy with the overall aim of ensuring good health for both the mother and the unborn child [1]. ANC services such as education, investigations, examination and treatment tends to contribute to the identification and prevention of pregnancy-related health problems [2]. Perhaps reporting for ANC in the first trimester may allow for early detection of pregnancy related complications and treatment. In fact, better pregnancy outcomes are greatly connected to provision and utilization of maternal health services [3]. Maternal health services essentially encompass assessment, education, treatment and prevention of pregnancy related health problems.

Globally, for the period of 2007–2014, 83% of pregnant women received antenatal care at least once [4]. Out of this, only 64% received up to the recommended four visits [4] suggesting the need for expansion in coverage of antenatal care. The high rates of ANC uptake reported in Africa is seen as a success story and appears to have a connection with the reduction in maternal mortality [3, 5]. ANC can be seen as an important activity that helps improve the health of pregnant women and ultimately leads to reduction in maternal morbidity and mortality.

Despite the shift in attention to focus ANC, there are health system challenges including lack of midwives as well as other relevant resources needed to deliver effective ANC services particularly in Africa [6]. In addition, the level of education, economic status, cultural beliefs, and other geographical barriers are known to influence the use of ANC among pregnant women in low-income countries. For instance, cultural and traditional beliefs about pregnancies to some extent influences the use of ANC among women in Nepal [7]. In Kenya, a review paper revealed that the barriers to ANC and delivery services include fear of testing HIV positive, unfriendly attitude of health staff, long waiting time and lack of transport [9]. Other Studies indicate that the barriers to ANC include lack of knowledge on the benefits of ANC, previous birthing experiences, male partner inability or unwillingness to support or accompany wives and loss of working hours [9–11]. In Ghana, Studies indicate that attending ANC is influenced by level of education thus those with formal education are likely to attend ANC [12, 13]. Also, women who are registered with the National Health Insurance Scheme and those living in urban areas are likely to attend ANC [12, 13].

According to the Annual Family Health Report, Ghana recorded approximately 87% ANC coverage in the year 2014 and the Upper East Region equally had a high (83.7%) ANC uptake [14]. However, the ANC uptake in Binduri district was 37.4% making it the district with the lowest ANC coverage in the region [14]. ANC coverage in Binduri district was 46.7% in the year 2015 and 47.6% in 2016 and this was the lowest in comparison to all the other districts in the Upper Region [19, 20]. The Ghana Health Services report for the year 2017 and 2018 still continue to show a slight decline in ANC coverage in the upper East Region of which Binduri is part [21, 22]. We therefore sought to explore womens' perspective on the determinants of ANC utilization in the Binduri district of Upper East Region of Ghana given the extreme low uptake of ANC services in the area.

## 2. Methods

### 2.1. Study Design

A qualitative approach using exploratory descriptive design was used. This design allows the researcher to explore the participants' perspective of the phenomenon under investigation [23].

### 2.2. Research Setting

The Binduri district is one of the eleven districts/municipalities in the Upper East Region of Ghana. The Binduri district was created out of the Bawku Municipality in the year 2012. The Binduri district is located approximately between latitudes 11^0^ 11^1^ and 10^0^ 40^1^N and longitude 0^0^18^1^W and 0^0^ 6^1^E in the north-eastern corner of the region. The district has a population of 61,576 representing 5.9% the total population of Upper East Region [15]. The population is entirely rural with males constituting 48.1% and females constituting 51.9% of the total population. The General Fertility Rate (GFR) is 97.5 births per 1000 women aged 15–49 years which is the third highest in the Upper East Region. About 5 in 10 (51.4%) of the population above age 12 years are married [15]. For females between the ages of 25–29 years, more than half (79.5%) are married compared to about 49.0% of males [15]. The Binduri district has two (2) health centers, one (1) clinic and seven (7) community-based health planning services (CHPS) compounds.

### 2.3. Target Population and Sampling Technique

The study was conducted among women residents in Binduri district who had given birth between 2014 and 2016 but did not attend ANC clinic. They were also women of age 18–44 years who could speak Kusaal or English and had the mental capacity to share their views on the issues raised. Purposive sampling technique was used to select women who met the inclusion criteria to share their perspective on the determinants of ANC uptake. Women recruited for the study were those who stayed within the district during period of January, 2014 and December, 2016 and delivered, yet did not attend ANC. They were also women who could speak Kusaal or English and voluntarily consented to participate in the study. Data saturation was reached by the time the 15^th^ participant was interviewed [16].

### 2.4. Data Collection Method

In the field, community health volunteers were identified and the purpose of the study explained to them. With the help of the community health volunteers, women who gave birth but did not receive antenatal care were identified. The principal researcher and community health volunteers then visited the identified women where the women were further screened to ensure they met the inclusion criteria and the purpose of the study explained to them. Those that agreed to participate in the study were given an option to schedule a suitable date and time for the interviews to be conducted. The interviews were done in the participants' homes. Individual face-to-face interviews were conducted in Kusaal and audio-recorded using a digital voice recorder. The kusaal audio-recorded interviews were transcribed in English based on the meaning of the statements. The transcripts were discussed with an expert in the kusaal language and confidentiality was ensured in the process. The women were asked open ended questions and probes used when necessary.

### 2.5. Data Processing and Analysis

The data was analysed using inductive thematic analysis. The audio-recorded interviews were transcribed verbatim. The audio-recorded information was compared to the transcribed data to ensure accuracy. The transcripts were then read through over and over to obtain a good grasp of the data. The key ideas were highlighted and notes of first impressions made alongside. These were labeled as initial codes which were sorted into themes and subthemes based on how differently they were related. The analysis was done individually by two of the authors (GTN and CAA). This was followed by a series of discussions between the two authors and the identified themes and subthemes agreed upon by these authors were used.

### 2.6. Ethical Considerations

Ethical clearance was obtained from Institutional Review Board of Noguchi Memorial Institute for Medical Research (NMIMR-IRB CPN 059/15-16). The purpose of the study was explained to the participants. Those who consented to participate were therefore recruited. Anonymity and confidentiality were observed as participants were given identification codes such as participant 1, 2, 3 etc., based on their chronologic enrolment into the study.

### 2.7. Methodological Rigour

Rigour of a qualitative study is the extent to which the identified meanings represent the perspectives of the participants accurately [17]. The four criteria for ensuring rigour according to [17] which include credibility, transferability, dependability and confirmability were ensured. To ensure credibility, a member check was done by getting some of the participants to confirm the accuracy of transcribed data and emerging themes as truly representing their experiences. A clear description of the procedure for participants' selection and detailed description of the research setting was done in order to enhance transferability. The method used for data collection, analysis and interpretation is also captured in the report for dependability. An audit trail comprising of field notes, audio recordings, analysis notes and coding details were also kept for confirmability.

## 3. Results

### 3.1. Demographics

Fifteen women aged 26–44 years were recruited for this study. All the women were married and in a monogamous union. All the women were farmers. Four of them had one child each, five had two children each and the rest had three or more children. Five of them were traditionalist, seven were Muslims and three were Christians. Three of them dropped out of school at the primary level but the rest said they had never received formal education. The participants were all Kusasi by tribe and could all speak Kusaal fluently.

### 3.2. Individual Factors

Most of the reasons accounting for failure to attend ANC were individual factors. They were issues that were peculiar to the individual participants and could hardly be generalised to others. The individual factors that were raised included financial constraints and health insurance related issues, excuses, perception of pregnancy and concentration on work.

### 3.3. Financial and Health Insurance Related Constraints

A number of the participants mentioned financial constraints as a barrier to the use of ANC during pregnancy. Although, the participants knew that pregnant women are entitled to a waiver when registering with the National Health Insurance Scheme (NHIS), the cost of traveling to the NHIS center was too huge for them to afford.

*“I know that ANC is free but you need health insurance paper to go to the hospital. The insurance people don't come to our community to register us and where they are is also far. You need money for transportation to the health insurance office which I didn't have.” (Participant 4).*

Some of the participants also stated that, the long queue one has to follow at the NHIS center for insurance renewal was also a disincentive that discouraged them.

*“What time will you leave here and get to the health insurance office and queue for the registration? The insurance office is always crowded with people and I can't get up at midnight to go there.” (Participants 15).*

Others also felt that it was often difficult to get their daily bread and therefore perceived the ANC as less important compared to involving themselves in productive ventures. Another explained how financial constraints kept her busy from attending antenatal.

*“Everything is about money but I don't have work. It is not easy for us. You have to spend time struggling around to see if you can get money for your ingredients to prepare soup.” (Participant 3).*

Participants also mentioned that if they go for ANC and unable to buy items that may be expected of them for delivery at the hospital, they could have issues with midwives.

*“If you go for ANC, they will tell you to buy certain things in preparation for the delivery at the clinic. Those items will require money to buy and I don't have money.” (Participants 15).*

Another issue raised by the women was that the health facilities within their catchment area were not equipped with ultrasound machines. Pregnant women are therefore generally referred to places where they can obtain this service.

*“Even though I knew the importance of attending clinic during pregnancy, I failed to do so because my friends who went were asked to go to a bigger hospital for a picture (scan) and this is in Bawku which is far. You cannot walk to Bawku. You need money for transport and to pay for the scan.” (Participant 1).*

Though participants were aware of the free coverage of ANC under NHIS, they were unsure if they will pay for services that were not available at the health centres within their community. This affected their readiness to go for ANC as explained below:

*“I know that if you are registered under the NHIS you will be treated free but if they refer you for a test in another hospital, I cannot tell if you will pay or not. And you know the village here every small thing they will say go to a bigger hospital meanwhile you don't not even have the money.” (Participant 12).*

### 3.4. Excuses

The women gave various reasons why they could not attend ANC during their pregnancy. Some of the reasons mentioned suggest that the women did not place much importance on ANC.

*“When I was about 2 months pregnant my father fell sick and I went to take care of him. He later died and by the time the funeral finished and I returned to my marital home I was already more than 5 months pregnant. I wanted to still report for the ANC but my friend told me that you can only report for ANC in the first 3 months of the pregnancy if not the midwife will insult you.” (Participant 8).*

No evidence of effects of not attending ANC was yet another reason for not attending ANC.

*“I have given birth to seven children and out of that number, I attended ANC for only one pregnancy. And whether you go for the ANC or not there is no difference when you are go into labour. You will go through the same labour pain.” (Participant 14).*

Some tried to blame their inability to attend ANC on lack of support for the care of the other children.

*“I already have 5 children and they are all small. I can't leave them alone and go anywhere. You know we stay alone and my husband travelled to kumasi to work.” (Participant 10).*

*“As a woman you have a lot of women work to do so being pregnant does not make you even free in a way. I will say I was just busy and could not find time.” (Participant 14).*

#### 3.5. Perception of Pregnancy

The routine checks done during ANC are to detect and intervene for deviations in pregnancy. Some participants rather reasoned that attending ANC is for pregnant women who are sick. This implies pregnant women who feel well may not need to attend ANC.

*“When I was pregnant I had no problem. I was not vomiting and not weak. I could go about my daily routine and therefore there was no reason to go to the hospital.” (Participant 8).*

Some actually thought that once they were active then there was no need.

*“Going to hospital is good but pregnancy is not sickness. I was fine and went about my normal activities.” (Participant 7).*

### 3.6. Concentration on Work

Participants in monogamous marriages felt that their inability to attend ANC was as a result of so much workload at home.

One participant explained.

*“I am the only wife of my husband and the work in this house is on me because my children are still young. They cannot even fetch water from the borehole, sweep or even cook.” (Participant 10).*

Some participants preferred to get busy with their farms.

*“During rainy season like this, you have to sow for your husband and work on your own rice so we are very busy. Everyone in the community is very busy and you cannot afford to be the only one who is not.” (Participant 3).*

Another participant added that:

*“I was pregnant during the rainy season and that is the time we do serious farming. It was difficult to make time to go for ANC.” (Participant 2).*

### 3.7. Health System Factors

The women indicated that attitude of nurses contributed to their inability to access ANC.

### 3.8. Perceived Nurses Attitude

The participants alleged that nurses do not treat clients well and this does not encourage them to attend ANC. The participants themselves did not have evidence of bad attitude of nurses during ANC since they did not attend. However, they drew inferences from what they heard from others and previous encounters with nurses.

*“We hear that the nurses are very rude. They don't respect the pregnant women and sometimes shout. I did not want to face such an embarrassment that is why I did not go to the hospital.” (Participant 5).*

*“A friend told me that a nurse ask whether it was she the nurse who made her pregnant and why she was frowning her face like that at her. You just sometimes don't know how to please the nurses so you just stay at home and avoid problems.” (Participant 4).*

A participant inferred from an incident that happened 2 years ago.

*“My first child had convulsion we rushed her to the health centre. The nurses were blaming me for no reason. Can you imagine the nurse even spoke about my dirty and smelling cloth? Since then I have not been there and thank God my children are healthy.” (Participant 7).*

## 4. Discussion

The study was conducted among women who had delivered but did not attend antenatal clinic during their pregnancy. Individual and health system related factors were responsible for nonattendance of ANC among our participants. In the Ghanaian health system, the National Health Insurance Scheme (NHIS) provides coverage for all registered members under the scheme but the government policy allows all pregnant women to register at no cost with the NHIS and access healthcare. Despite this provision by the government of Ghana, some of the participants explained that cost of transport and delays at the NHIS were factors that hindered their ability to go for the registration. Similarly, studies have suggested that even though health insurance ensures the provision of free ANC, secondary cost may hinder utilisation [12, 18]. Reduction of poverty is therefore an essential factor to ensure that women of rural background are resourced to be able to attend ANC.

The study findings suggest that most of the participants did not know the essence of attending ANC so when questioned they only came up with excuses. Some excuses for not attending ANC were being busy with house chores and attending funerals. Considering the medical importance of ANC, one will expect pregnant women to make time to attend ANC hence excuses such as those mentioned by participants in this study may suggest inadequate knowledge on ANC. A study among health staff in antenatal clinic indicated lack of knowledge on ANC as a barrier for uptake of ANC [10].

Some of the participants mentioned farming activities as a barrier to attending ANC. This point to the fact that economic activities were given much priority as compared to ANC. Finlayson and Downe [11] also established that even where women recognize the benefits of ANC, potential loss of crucial family resources can interfere with uptake. The participants in this study were all from farming communities and engaged in farming as their main source of livelihood hence time lost is crucial during farming activities. The study finding could also mean the women in the present study did not have enough economic support from their husbands to enable them to make time for ANC.

Another stand expressed by some of the participants was that “pregnancy is not sickness”. With this ideology the participants did not see why they should attend ANC because they knew they were not sick and the fact they were pregnant did mean they should go to a health facility. In consonance Finlayson and Downe [11] revealed that participants viewed pregnancy as a healthy physical state and saw little reason to visit health professionals when there was no perceived threat to their well-being. ANC is very important to prevent pregnancy-related health problems through education, investigations, examination and treatment among others [3, 2] Pregnant women with the ideology that they are not sick will not benefit from early detection and prevention of pregnancy related complications since they are only likely to report at the hospital on condition that they are sick.

The women mentioned attitude of nurses as one of the factors why they chose not to attend ANC during their period of pregnancy. The inability to treat patients with respect has far reaching effect of preventing other clients from coming for the ANC as revealed in this study. Yadufashije and Samuel [9] reported that unprofessional practices, attitudes and behaviors of health workers may decrease utilization of ANC. In the present study, though the women did not attend ANC some drew inferences from previous encounters with nurses while others relied on information they received from those who attended ANC.

Inadequate resources at health facilities in the communities where the study was conducted was yet another issue raised by the participants for their non-attendance of ANC. The participants who were generally of low income bracket intimated that attending ANC at the communities is likely to end up being referred for laboratory investigations and ultrasonography which will come with financial cost. This finding corroborates that of Asamoah et al. [13] which stated that women who are not educated, poor and stay in rural areas are less likely to attend ANC. In this study the limited financial capabilities of the women to carry out any requested investigation outside the health facilities in the communities was deterrent to ANC attendance.

### 4.1. Strengths and Weaknesses of the Study

The study explored the determinants of ANC uptake among women who have delivered without attending ANC hence the findings will set the background for others studies on this phenomenon.

The study did not include health workers, husbands and opinion leaders in the communities hence do not present a multidimensional perspective of the issue under investigation. The small number of participants used for the study does not also allow for generalization of the findings.

## 5. Conclusion

The study identified individual and health system factors that were responsible for the non utilisation of ANC. The National Health Insurance Scheme which forms the basis for free ANC should be made more accessible through establishment of registration centres in all communities. There should also be intensive public education on the health benefits of attending ANC so that pregnant women will make it a priority. Community health volunteers should also be given incentives to identify pregnant women who do not attend ANC so that they could be educated and encouraged to attend ANC.

## Data Availability

The data used to support the findings of this study are available from the corresponding author upon request.

## Abbreviations

ANC: Antenatal care
NHIS: National Health Insurance Scheme.
